# The Challenge of Living On: Psychopathology and Its Mediating Influence on the Readjustment of Former Child Soldiers

**DOI:** 10.1371/journal.pone.0102786

**Published:** 2014-07-23

**Authors:** Verena Ertl, Anett Pfeiffer, Elisabeth Schauer-Kaiser, Thomas Elbert, Frank Neuner

**Affiliations:** 1 Department of Psychology, Clinical Psychology and Psychotherapy, Bielefeld University, Bielefeld, Germany; 2 vivo international, Konstanz, Germany; 3 Clinical Psychology and Neuropsychology, Department of Psychology, University of Konstanz, Konstanz, Germany; 4 Department of Psychology, Clinical Psychology and Neuropsychology, University of Konstanz, Konstanz, Germany; University of California, San Francisco, United States of America

## Abstract

Current civil wars are characterized by the increasing involvement of civilian populations and the systematic employment of child soldiers. An example of such wars was the conflict in Northern Uganda, where the war-affected population is still challenged by the reintegration of formerly abducted children and youths. A cross-sectional, population-based survey, using a multistage cluster sampling approach of 1,113 Northern Ugandans aged between 12 and 25 in camps for internally displaced persons and locally validated instruments was conducted to assess symptoms and diagnoses of Posttraumatic Stress Disorder (PTSD) and probable Depression in war-affected, as well as formerly abducted individuals. Further objectives were to determine predictors of psychopathology and to relate indicators of maladjustment (i.e., impairments in daily and community functioning, somatic complaints, suicidality, aggressiveness and discrimination) to abduction, level of exposure to violence and psychopathology. 43% of the sample reported abduction by the rebel army. Exposure to violence among this group was higher than for non-abducted youths (*t* = 28.05; *p*<.001). PTSD point prevalence rates were 25% among former child soldiers and 7% among the comparison group. High suicidal ideation was present in 16% and 6% respectively. A higher amount of experienced and witnessed event-types (*β* = . 32. *p*<.001), loss of first-degree relatives (*β* = .13. *p*<.001) and the number of event-types involving forced perpetration (*β* = .23. *p*<.001) were identified as risk factors of PTSD symptoms in former child soldiers. The associations between abductee-status and indicators of maladjustment were fully mediated by level of trauma exposure and psychopathology. Results show that child soldiering and its psychological sequelae affect a substantial proportion of children and youths. After release or flight, their readjustment depends at least partly on their level of mental traumatization.

## Introduction

Currently more than 50 parties in conflict-areas around the globe recruit children and an estimated 250.000 children under the age of 18 are actively involved as child soldiers in hostilities in at least 14 countries [1], [2]. One example of these conflicts was the civil war in Northern Uganda, which is still continuing on territories of Southern Sudan, the Central African Republic and DR Congo. The forces of the local rebel group Lord's Resistance Army (LRA) almost exclusively consist of abducted children. Within two decades of fighting, an estimated 75.000 Ugandan children and young adults had been abducted and were forced to serve the LRA as porters, combatants and sexual slaves [3]. In order to protect the local communities, virtually the entire Northern Ugandan population, approximately 1.8 million people, were resettled in camps for internally displaced persons (IDP camps), which they were reluctant to leave after a gradual calming of the conflict in 2008 [4]. Throughout the fighting and thereafter, the communities have been confronted with large numbers of formerly abducted children who have tried to rejoin their families after their rescue, flight or release. However, the reintegration of these children and adolescents is challenging up to now due to fear within the community concerning their alienated children, who had often been forced to commit atrocities. In addition behavioral disturbances caused by extensive exposure to violence, suspiciousness on both sides and the delay in education interfere with successful readjustment to civil life and healthy development [5]–[8].

A child soldier has been defined as “any person below 18 years of age who is or who has been recruited or used by an armed force or armed group in any capacity, including but not limited to children, boys, and girls used as fighters, cooks, porters, messengers, spies or for sexual purposes.” [9]. This definition illustrates that child soldiers are a heterogeneous group with respect to their roles and the duration of their recruitment. Nevertheless, almost all abducted children share frequent exposure to severe stress, which may contribute to psychological disorders. In recent years some studies reported exceptionally high rates of psychological disorders in former child soldiers that exceeded those of war-affected comparison subjects who had not been recruited [10]–[17]. For instance, a PTSD point prevalence rate of 55% was found among former child soldiers in Nepal [12]. Related to the conflict in Northern Uganda estimations ranged from 27% among former child soldiers recruited from a rehabilitation centre for child soldiers and a local college [16] to 84% in a community sample of adults who reported a history of abduction [15]. Rates of probable Depression in these samples varied between 20% and 88% [13], [16]. However, these figures have to be interpreted with care, as in most studies the examined populations were small and highly selective. In addition, diagnostic decisions were often made by assessors who had limited experience and training and relied on instruments that had not been validated in the respective language. Diagnoses were based on cut-off scores that had been derived from research in other populations. All of these factors can lead to considerable errors in estimations of prevalence [18], [19].

To date, studies on psychopathology in former child soldiers have failed to consistently identify patterns of risk and protective factors. The level of trauma exposure during captivity was found to predict PTSD in some [12], [14], [20], [21], but not in other studies [10], [11]. Neither the duration of abduction [10]–[12], [14], nor other variables related to rebel-experiences, like age at abduction [11], [12] and type of recruitment, as well as military role [12] were found to be independent predictors of PTSD. Likewise, indicators of the post-deployment phase, i.e. mode of return, time passed since return, rehabilitation provisions and traditional cleansing rituals did not influence PTSD or Depression [12]–[14]. Social support in the community upon return failed to emerge as a protective factor against PTSD [11], [14], whereas perceived spiritual support seemed to lower the risk of PTSD, as did higher economic status [11], [12]. Indicators of successful social reintegration, including high acceptance [7], [14], a weak continuous association with the rebel group [12], low perceived discrimination [8], low revenge motivation [10], [11] and the non-acceptance of violent conflict resolution strategies [15] were significantly related to mental health. While the causality of the relationships remains unresolved, these associations indicate that psychopathology might be interrelated with successful reintegration of former child soldiers.

Building on this prior research the objectives of our study were to estimate point prevalence rates of PTSD, probable Depression and suicidal ideation in a population-based survey of 12 to 25 year old Northern Ugandans, using validated assessment instruments and to compare exposure to traumatic events (as victims and perpetrators) and mental health outcomes in former child soldiers versus war-affected youths. In addition, we aimed to identify probable risk and protective factors of mental health outcomes in this sample. Finally, we hypothesized that former child soldiers would show higher levels of maladjustment, indicated by problems with physical health, functioning, suicidal ideation, perceived stigmatization and aggressiveness than their non-abducted peers. Based on the assumption of a dose-effect of trauma exposure (e.g. [12], [14], [22]–[27]) we postulated that the negative outcome of child soldiering would be at least partly mediated by trauma exposure and psychopathology.

## Methods

### Sample Selection and Participants

During July 2007 and April 2008 we carried out a cross-sectional, population-based survey in three different Acholi regions of Northern Uganda using a cluster sampling approach. The study included individuals between 12 and 25 years in order to focus on the generation of Northern Ugandans who were most severely affected by abduction and child soldiering [28]. As there was neither any reliable census nor previous household survey data of the Northern Ugandan population available, we used a pragmatic sampling approach based on random selection of individuals and households within purposely chosen study areas. The three survey areas Anaka, Awer, and Padibe were chosen according to their degrees of war-exposure and distance from the largest town in Northern Uganda, Gulu. While Awer represents the relatively safe area close to Gulu, Padibe is at a long distance and was more affected by the war. Finally, Anaka was chosen to represent the rural areas with most documented rebel activity. In order to reflect the estimated ratio of Northern Ugandan civilians living in areas with varying war-exposure, we approached 357 households in Awer, 514 in Padibe and 572 in Anaka. Interviews were carried out in all IDP-camps and new settlement sites within the study areas. Each location was sub-divided into zones of approximately equal household and population size. Screening teams were assigned to these zones and randomly chose a sampling direction by spinning a pen from the zone centre. Every sixth household was chosen as the target household until the end of the assigned zone was reached. A new direction was then chosen and the procedure repeated.

Within each selected household all adolescents and young adults between 12 and 25 who were sleeping and eating in the household were listed and the screeners selected the target person according to computer generated random-digit lists for different household sizes. 130 households had no member in the targeted age-range, 31 chosen individuals were not interviewed due to acute alcohol intoxication, mental retardation or psychotic features, 150 individuals were not interviewed because there were three or more failed recruitment attempts and 19 chose not to participate.

Characteristics of the resulting sample are summarized in Table 1. The sample consisted of more females (*n* = 693) than males (*n* = 420), which is likely to be due to the fact that more males had been abducted and killed in the target age group. 474 individuals reported a history of abduction (*n* = 208 males and *n* = 266 females), with the duration of abduction ranging from several hours to more than ten years (mean = 6.22 months, median = 0.47 months). According to the Paris Principles [9] we refer to these children as former child soldiers, although only 47% of them were abducted for one month or longer. The average age at first abduction was 11 years (*SD* = 3.72). The vast majority of males and females were of Christian denomination (98.1% and 99.4%). Despite high rates of alcohol abuse and dependency in Northern Ugandan men generally [13], [29], [30] substance consumption was low in this sample of young people, suggesting that excessive consumption, abuse, dependency and other alcohol-related problems start at a later age. A strong association between alcohol abuse and age has been reported consistently in studies with Northern Ugandan samples [5], [7], [29]. Males and former child soldiers in the present study reported significantly more alcohol consumption (5.5% versus 2.7%; χ^2^ = 5.39; *p* = .02 and 5.7% versus 2.4%; χ^2^ = 8.40; *p* = .004 respectively) and sniffed petrol or glue more often than females and non-abducted individuals (1.0% versus 0.0%; χ^2^ = 6.62; *p* = .01 and 0.8% versus 0.0%; χ^2^ = 5.41; *p* = .02 respectively). As substance abuse did not turn out to be a significant issue in our sample, indicators of drug consumption were not used for further analysis.

**Table 1:** Characteristics of the Sample by Abduction Status (n = 1113).

	former child soldiers (n = 474)	war-affected youths (n = 639)	Statistic	p-value
Males, No. (%)	208 (43.88)	212 (33.18)	χ^2^ = 13.27	<.001
Age, mean (SD)	18.79 (3.76)	17.20 (3.79)	t = 6.92	<.001
Marital Status, No. (%)				
single	294 (62.03)	459 (71.83)		
married/cohabiting	163 (34.39)	164 (25.67)		
separated	15 (3.16)	13 (2.03)	χ^2^ = 12.31	.006
widowed	2 (0.42)	3 (0.47)		
Household members, mean (SD)	7.31 (3.40)	7.28 (3.31)	t = 0.16	.88
Main source of food, No. (%)				
agriculture	186 (39.24)	282 (44.13)		
food aid	191 (40.30)	231 (36.15)		
barter	2 (0.18)	0 (0.00)	LRχ^2^ = 6.21^a^	.10
market	95 (20.04)	126 (19.72)		
Economic Status per Person, mean (SD)^b^	51.95 (34.91)	60.96 (42.39)	Z = 3.86^c^	<.001
Highest level of education, No. (%)				
no schooling	32 (6.75)	30 (4.69)		
some primary	344 (72.57)	465 (72.77)		
completed primary	32 (6.75)	44 (6.89)		
vocational training	9 (1.90)	9 (1.41)		
some secondary	49 (10.34)	80 (12.52)	LRχ^2^ = 10.32^a^	.17
completed secondary	8 (1.69)	6 (0.94)		
completed advance level	0 (0.00)	4 (0.63)		
completed university	0 (0.00)	1 (0.16)		
Displacement, No. (%)	464 (97.89)	607 (94.99)	χ^2^ = 6.30	.01

*Notes.*

a likelihood ratio χ^2^.

b in Euro.

c nonparametric Mann-Whitney-U-Test.

### Measures

Luo (local language in Northern Uganda) versions of all instruments were created using a translation and blind back translation procedure. Recommendations for cultural adaption, ensuring conceptual, functional and semantic equivalence were considered [31].

#### Sociodemography

The first part of the screening interview consisted of sociodemographic questions about individual and household characteristics. Year of birth, gender, marital status, ethnicity, religious denomination and practice, level of education and history of displacement and abduction were recorded for each participant. The assessed household characteristics were household size, source of feeding and household possessions (sum of assets in possession of the participant's household weighted by the market price in local currency).

#### Trauma exposure

The Violence, War and Abduction Exposure Scale (VWAES) is a 34 item checklist of potentially traumatic events that was developed especially for use in the Northern Ugandan context, based on in-depth interviews with 30 former child soldiers. It consists of 18 general event types, adapted from the Clinician-Administered PTSD Scale ([CAPS]; [32]) event checklist, 6 LRA-specific event types that capture events related to the rebel army (e.g. “Have you ever been forced to eat human flesh by the LRA?”), 6 forced perpetration event types (e.g. “Have you ever been forced to kill someone by the LRA?”), 4 event types related to family violence, and the possibility to document 1 other event type experienced or witnessed (over-all range, 0–36).

#### PTSD

The Posttraumatic Stress Diagnostic Scale ([PDS]; [33]) has been widely used for the assessment of PTSD and provides measures of overall and subscale symptom severity. It consists of 17 items reflecting the core PTSD criteria of reexperiencing, avoidance and hyperarousal according to the DSM IV [34]. Each item can be scored on a 4-point scale (over-all range, 0–51). A previous validation study in the Acholi population [18] showed that our local version of the PDS had very good internal consistency (Cronbach's α = .89) and a good correspondence with expert diagnoses based on the CAPS as gold standard (sensitivity = .82, specificity = .76 and κ = .54).

#### Drug consumption

Alcohol consumption and the sniffing of glue or petrol were assessed with two questions concerning the time period of four weeks prior to the interview including frequency ratings.

#### Depression

Symptoms of Depression were assessed with the 15-item Depression section (DHSCL) of the Hopkins Symptom Checklist [35] which focuses on the perceived severity of symptoms during the week prior to the interview. Answers are coded on a 4-point likert scale. The DHSCL was chosen because it has been extensively used for the assessment of symptoms of Depression across a wide variety of cultures (e.g. [36], [37]) including several East African populations (e.g. [25], [26], [38]). Although reasonably good psychometric properties and validity have been reported elsewhere [39], [40] and internal consistency of the Acholi version of the DHSCL was very good (Cronbach's α = .89) in our validation study, it did not perform satisfyingly as a diagnostic tool (sensitivity = .50, specificity = .83 and κ = .32) [14]. However, since it proved to be valuable in grading symptom severity, we applied this instrument to determine symptoms of Depression.

#### Maladjustment

Following the findings of interviews with key informants (including community elders and representatives of a child soldiers' parents organization) as well as in-depth interviews with former child soldiers, we identified different indicators of successful integration and maladjustment in the community. When available, we chose appropriate standardized instruments to assess these constructs (stigmatization, aggressiveness, PDS functioning scale, suicidality). In addition, specific instruments for the assessment of dysfunction in the community (LFS) and for physical complaints were developed for this study. These six dimensions of impaired adjustment were aggregated to form one measure of maladjustment.

#### Illnesses

Current physical health status was assessed by asking the participants whether illnesses or medical conditions typically occurring in the region (e.g. Malaria, Diarrhea, etc.) were present within four weeks prior to the screening. The score of impaired physical health was generated by summing the types of medical conditions present (over-all range, 0–12).

#### Suicidality

Suicide risk was assessed with the module C of the Mini International Neuropsychiatric Interview (English Version 5.0.0; [41]). The MINI has shown reliable and valid results when administered by trained lay interviewers [42]. Current suicidal risk and risk levels were determined by adding the standard numeric weights of the 6 items as specified by the instrument. Participants were categorized into the risk levels low (range, 1–5), medium (range, 6–9), and high (range, 10–33) according to these weights.

#### Impairment of Functioning

In order to gain more detailed knowledge about functional impairment in specific daily routines a local measure of functioning was developed, since other detailed instruments contain too many culture-bound questions. The process of developing the Luo Functioning Scale (LFS) followed an approach described by Bolton and Tang [43]. Fifty local informants were asked to list tasks that teenage-girls/women and teenage boys/men must do regularly to care for themselves, for their family and for their community. The nine most frequently mentioned tasks (three per category) were compiled in separate functioning questionnaires for females and males. A tenth question about sexual interest/activity was added. Respondents were asked about the degree of difficulty they experienced in completing the tasks or activities in the past month. Answers were rated on a 5-point likert scale ranging from 0, “none” to 4, “often can't do task”. The causes of difficulties were documented for each item rated at least 1, and causes not referring to physical, mental or emotional problems (e.g. lack of financial means) led to coding the item 0. While piloting the scale it became apparent that some of the items were not suitable for children below the age of 16. Therefore shortened versions were used for these participants, consisting of 6 age appropriate items for females and 4 items for males respectively (score-range, 0–4). The PDS contains eight items that relate to impaired functioning. The sumscore of these items was used as an additional indicator for impairment of functioning (score-range, 0–8).

#### Stigmatization

The Perceived Stigmatization Questionnaire ([PSQ]; [44]) was shortened to a 12-item version representing the 2 factors “confused, staring and hostile behavior” (e.g. “People act surprised or startled when they see me.” “People call me names.”) and “absence of friendly behavior” (e.g. “People treat me with respect.” [reverse coded]). Respondents' answers concerning the frequency of stigmatizing behavior during the 4 weeks prior to the screening were coded on a 5-point Likert scale with 0 representing “never” to 4 representing “always”. One item was deleted due to frequent misunderstandings. The adapted version of the PSQ revealed good internal consistency of the scale scores (α = .83; score range, 0–4).

#### Aggressiveness

Aggressiveness was assessed via a shortened version of the Aggression Questionnaire by Buss and Perry [45]. The instrument allows for the assessment of four subtypes of aggression, (physical aggression [e.g. “Once in a while I can't control the urge to strike another person.”], verbal aggression [e.g. “I can't help getting into arguments when people disagree with me.”], anger [e.g. “I have trouble controlling my temper.”] and hostility [e.g. “Usually everybody is against me”]). Total and subscale scores can be calculated. Participants' answers to 16 statements were coded on a 5-point Likert scale ranging from 0, “extremely uncharacteristic of me” to 4, “extremely characteristic of me. A Cronbach's α of α = .81 indicated good internal consistency of the modified scale version (score range, 0–4).

### Procedure

Interviews were carried out by 18 local screeners who had received six weeks of training, which ended with a theoretical and practical exam, as well as an additional four weeks of practical training by a team of international clinicians and researchers. Screeners were trained in relevant psychological concepts to the extent that they could flexibly handle clinical tools and judge the presence or absence of symptoms autonomously. The relatively long training period was necessary to ensure that the team of interviewers was proficient in diagnostics and the individualized adaptation of screening instruments in order to cope with the high variance of education and capacity among the respondents.

#### Ethics Statement

Screening interviews were started after a comprehensive explanation of the study was provided and written informed consent was obtained. Where the participant was under age, a guardian was additionally informed about the study and consent was obtained from both, participant and guardian in written form (signature or fingerprint). The study was approved by the Ethics Committee of the University of Konstanz, Germany as well as by the Ethics Committee of the Mbarara University of Science and Technology, Uganda.

### Data Analyses

As the sample was selected as being representative with respect to conflict exposure we used unweighted data for determining the level of trauma exposure and point prevalence rates of PTSD. We compared subjects with a history of abduction to non-abducted comparison subjects with regard to demographic variables, trauma exposure, psychopathology and various indicators of maladjustment.

In order to identify possible predictors of mental health status for the Northern Ugandan war-affected youths multiple regression models were applied using continuous measures of PTSD and Depression as dependent variables. To allow the determination of predictors related to variances in experiences connected to captivity separate analyses were carried out for the former child soldiers and the full sample. Continuous as well as dummy-coded nominal variables entered the models as potential predictor variables.

To study the relationship between abduction, trauma exposure, psychopathology and indicators of impaired readjustment, we calculated multiple step multiple mediator models. Using this approach we tested whether the relationship between abduction and outcome was mediated by psychopathology, which, in turn, depends on traumatic exposure. Mediation models were calculated separately for males and females, since gender differences concerning psychopathology as well as related gender-specific patterns of traumatic experiences (e.g. concerning sexual violence) have been consistently reported not only in Western societies, but also in former child soldiers (e.g. [5], [12], [17]). For the mediation models, we used composite measures of psychopathology (mean of z-transformed levels of PTSD and Depression scores) and maladjustment (mean of z-transformed scores of stigmatization, aggressiveness, PDS functioning, suicidality, dysfunction in the community (LFS) and physical complaints). After calculating models for these composite variables, we calculated a series of exploratory in-depth models for the single indicators of impaired adjustment, separately for symptoms of Depression and PTSD. Multiple step multiple mediation models not only allow simultaneous mediation by multiple variables, like single step multiple mediation models, but also take dependency of the mediators into account. Mediation models were calculated according to the procedure described by Hayes, Preacher, and Myers [46]. Direct and indirect effects of abduction on measures of maladjustment were estimated using a set of ordinary least squares regressions. Standardized estimates of the resulting path coefficients, as well as tests of significance for each path were calculated using three regressions (one for each of the mediators as outcomes and one for the chosen measure of impaired readjustment as outcome). We used bootstrapping based on 5000 bootstrap samples to infer statistical significance on the level α = .05. Findings from various simulations show that bootstrapping has greater statistical power than the product of coefficients strategy for detecting indirect effects when they are present [47]–[49]. Since indirect effect estimates were calculated based on bootstrapping, the repeated process of sampling 1113 cases randomly with replacement 5000 times also eliminated cluster effects that might have been present due to similarities within same locations. Data analyses were carried out with JMP version 8.0 (SAS Institute Inc., Cary, North Carolina) and SPSS version 18 (SPSS Inc, Chicago, Illinois).

## Results

### Descriptive Statistics and Prediction of Psychopathology

**Figure 1:**
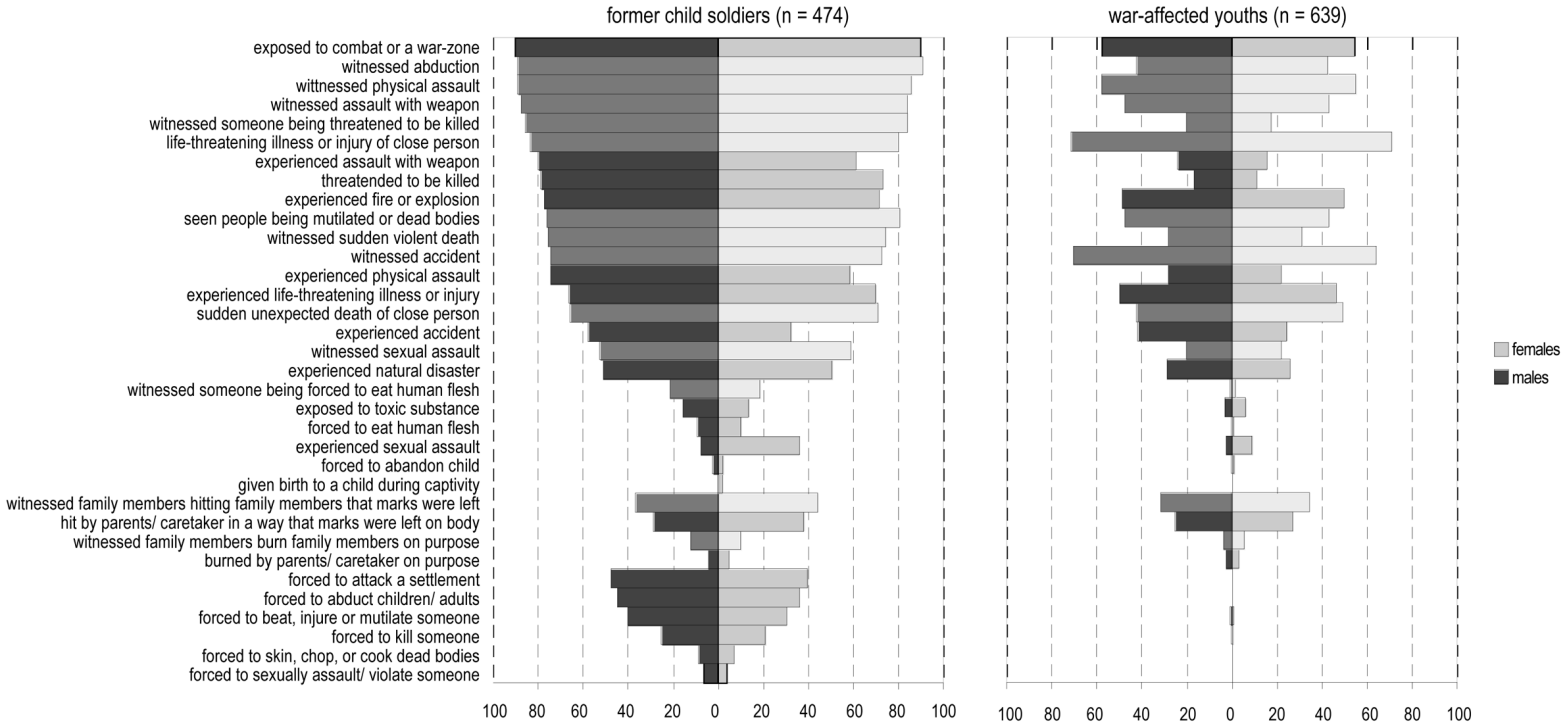
Frequencies of Traumatic Event-types for Former Child Soldiers and War-affected Youths by Gender. *Note.* Lighter shades of grey within gender indicate witnessed event-types, darker shades of grey indicate experienced event-types.

Exposure to traumatic event-types was high, with only 2% of the 1113 respondents reporting no confrontation with potentially traumatizing events. Event-types with forced perpetration were reported by 57% of the subsample of formerly abducted youths. Figure 1 displays the frequencies of single event-types for the formerly abducted, as well as for the non-abducted comparison subgroup split by gender.

The PDS provided information on the worst event indicated by the subjects. The most severe events, as indicated by their relative probability of being singled out as the worst event among the events reported, were being forced to kill (61%), followed by witnessed killing (34%), sudden unexpected death of a close person (22%), seeing somebody being mutilated or dead bodies (18%) and sexual assault (12%). Worst events were experienced early in life, at a mean age of 12.5 years (*SD* = 4.26). Point prevalence rates of PTSD were 7% for non-abducted individuals, 25% for former child soldiers and 15% for the total sample. Rates of suicidal ideation were 19% (high risk: 6%) for non-abducted respondents, 34% (high risk: 16%) for former child soldiers and 25% for the total sample (high risk: 10%). Table 2 shows the characteristics of the subgroup of former child soldiers in comparison to the non-abducted individuals concerning traumatic event-load, psychopathology and the indicators of maladjustment. Table 3 displays the results of the multiple regression analyses on PTSD and Depression symptoms. Trauma exposure predicted symptoms of PTSD and depression. Female gender and older age were independent predictors of symptoms of depression. Older age and economic status predicted symptoms of PTSD in the total sample of war-affected youths. Actively practicing religion was independently associated with symptoms of Depression, but not PTSD in both, former child soldiers and the total sample of war-affected youths. The number of first-degree family members that died due to the LRA insurgency independently predicted PTSD and Depression symptom load beyond traumatic exposure and socio-demographic variables.

**Table 2:** Traumatic Event-load, Psychopathology and Indicators of Maladjustment by Abduction Status (n = 1113).

	former child soldiers (n = 474)	war-affected youths (n = 639)	Statistic	p-value
Event-load total, mean (SD)^a^	16.47 (5.70)	7.90 (4.00)	t = 28.05^b^	<.001
Event-load experienced^c^	6.72 (2.51)	3.27 (1.98)	t = 24.75^b^	<.001
Event-load witnessed^d^	7.32 (2.06)	3.95 (2.15)	t = 26.30	<.001
Event-load events with forced perpetration^e^	1.53 (1.71)	0.01 (0.13)	Z = 21.35^f^	<.001
Event-load severe domestic violence^g^	0.90 (0.96)	0.67 (0.84)	Z = 3.91^f^	<.001
PTSD Symptoms, mean (SD)^h^	10.25 (8.46)	5.15 (6.58)	Z = 10.94^f^	<.001
Depression Symptoms, mean (SD)^g^	1.97 (0.64)	1.64 (0.58)	Z = 9.00^f^	<.001
Functional Impairment (PDS), mean (SD)^i^	2.63 (2.55)	1.22 (1.97)	Z = 10.42^f^	<.001
Functional Impairment (LFS), mean (SD)^g^	0.27 (0.47)	0.15 (0.32)	Z = 5.93^f^	<.001
Suicidal Ideation, No. (%)				
low	49 (10.34)	48 (7.51)		
medium	36 (7.59)	36 (5.63)	χ^2^ = 38.19	<.001
high	74 (15.61)	37 (5.79)		
Physical complaints, mean (SD)^j^	5.19 (2.32)	4.57 (2.19)	t = 4.54	<.001
Aggressiveness, mean (SD)^g^	1.54 (0.83)	1.20 (0.71)	t = 7.20	<.001
Stigmatization, mean (SD)^g^	0.75 (0.72)	0.53 (0.59)	Z = 4.88^f^	<.001

*Notes.*

a maximum score = 36.

b t-test for unequal variances.

c maximum score = 15.

d maximum score = 11.

e maximum score = 6.

f nonparametric Mann-Whitney-U-Test.

g maximum score = 4.

h maximum score = 51.

i maximum score = 8.

j maximum score = 12.

**Table 3:** Standardized Beta Coefficients Resulting from Multiple Regression Models on PTSD and Depression Symptoms.

	Symptoms of PTSD				Symptoms of Depression			
	former child soldiers (n = 474)^a^		whole sample (n = 1113)^b^		former child soldiers (n = 474)^c^		whole sample (n = 1113)^d^	
Predictor	β	r	β	r	β	r	β	r
Gender (male)	−.06	.01	−.04	−.01	−.18***	−.14**	−.11***	−.08**
Age	.02	−.02	.08**	.09**	.21***	.17***	.20***	.24***
Location (Anaka>Padibe>Awer)		.16***		.12***		.11*		.11***
Padibe Area – Awer Area	−.01		.00		−.08		−.01	
Anaka Area – Padibe Area	.18***		.15***		.20***		.18***	
Economic Status	−.03	−.07	−.07*	−.10**	−.09*	−.10*	−.05	−.08**
Abduction duration	.01	.34***			−.11**	.19***		
Regular Praying	−.07	−.05	−.03	.01	−.12**	−.07	−.10***	−.05
1^st^ grade family members died due to war	.13***	.19***	.12***	.20***	.09*	.18***	.13***	.21***
Event-types (experienced, witnessed, domestic)	.32***	.51***	.31***	.34***	.24***	.43***	.28***	.34***
Event-types with forced perpetration	.23***	.43***	−.04	.02	.30***	.36***	.01	.06*

*Notes.*

a Full model's adjusted *R* ^2^ = .30; *F* (10, 463) = 21.58, *p*<.0001.

b Full model's adjusted *R* ^2^ = .15; *F* (9,1103) = 22.89, *p*<.0001:

c Full model's adjusted *R* ^2^ = .31; *F* (10, 463) = 22.06, *p*<.0001.

d Full model's adjusted *R* ^2^ = .19; *F* (9, 1103) = 30.50, *p*<.0001.

Zero-order correlations are represented by Spearman's ρ for continuous predictor variables and point-biserial correlations for dichotomous predictor variables. Symbols indicate significance:

*p<.05.

**p<.01.

***p<.001.

### Mediation of impaired Readjustment

**Figure 2:**
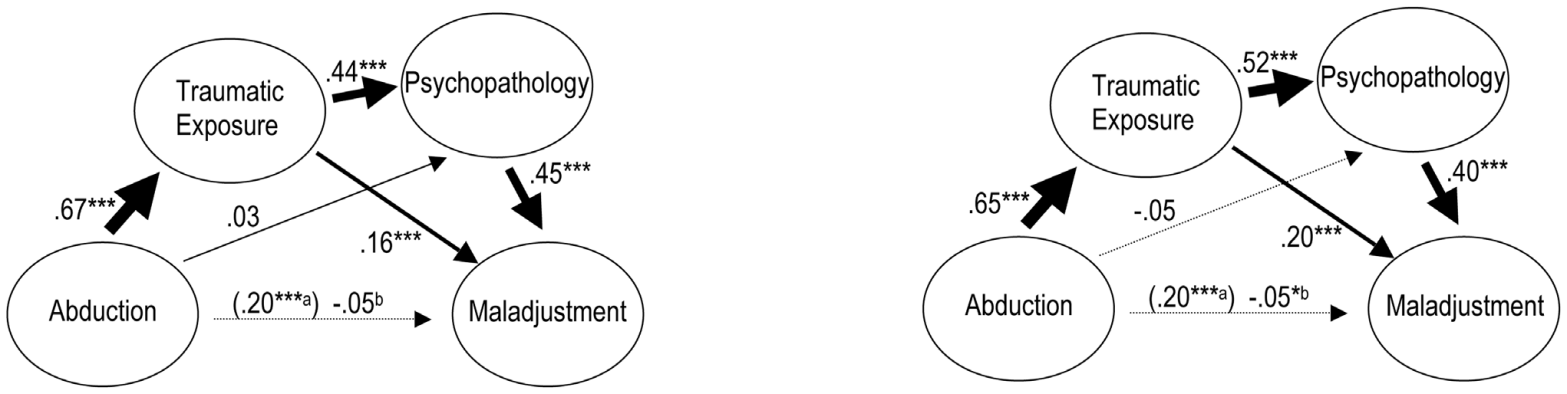
Multiple Step Multiple Mediation Models for Composite Measures of Psychopathology and Maladjustment for males (left) and females (right). *Notes*. ^a^ Standardized regression coefficients predicting maladjustment by abduction, neglecting the mediating influences of traumatic exposure and psychopathology. ^b^ Taking mediation into account the direct effect of abduction on maladjustment becomes irrelevant. Symbols indicate significance: * *p*<.05. **p<.01. *** *p*<.0001.

The multiple step multiple mediation models shown in Figure 2 are based on the composite measures for psychopathology and maladjustment split by gender. The models were very similar for both sexes. Abduction was a highly significant and strong predictor of trauma exposure, which, in turn, strongly predicted psychopathology when controlling for the effects of abduction, whereas the influence of abduction on psychopathology disappeared when adjusting for traumatic exposure. Psychopathology was the strongest predictor of maladjustment in both sexes when controlling for the effects of abduction and trauma exposure. However, a significant independent contribution of trauma exposure to maladjustment remained, even when controlling for psychopathology and abduction. Controlling for both mediators - traumatic exposure and psychopathology - the positive influence of abduction on maladjustment disappeared completely.

To establish how much of the effect of abduction on the extent of maladjustment is direct and how much is indirect via the two mediators, the relevant beta weights of the paths linking abduction to maladjustment via the mediators were multiplied to provide estimates of the three specific indirect effects. The bootstrapping procedure yielded 95% confidence intervals and thus allowed to infer the significance of the specific indirect effects and the total indirect effect, i.e. whether mediation according to our model was present or not.

The first specific indirect effect, representing the effect of abduction on maladjustment through traumatic exposure was .11 for males (*CI* = .07, .15) and .13 for females (*CI* = .10, .17). The second specific indirect effect of abduction on maladjustment via psychopathology reached a value of .01 for males (*CI* = −.03, .05) and −.02 for females (*CI* = −.05, .01). Finally, the third specific indirect effect representing the path from abduction, via trauma exposure and psychopathology to maladjustment was .13 for males (*CI* = .09, .17) and .14 for females (*CI* = .11, .17). Overall the single indirect effects added up to total indirect effects of .25 for males (*CI* = .20, .31), as well as females (*CI* = .20, .30). For both genders the relationship between abduction and the extent of maladjustment was fully mediated by traumatic exposure and psychopathology in dependency on traumatic exposure.

To explore model path estimates (standardized betas) and effect estimates of multiple step multiple mediation models that were more specific and to estimate the robustness of the mediation effect, we calculated a series of mediation models, separately for PTSD and Depression symptoms and the six original components of the composite maladjustment variable (available upon request). Overall, all specific models were consistent with the general model. The direct effects of abduction on the different measures of maladjustment (impaired functioning (PDS and LFS), suicidality, physical complaints, aggressiveness and stigmatization) were significant and ranged between β = .12 and β = .39. For both genders total indirect effects were significant, ranging from .14 (in the model for males with impaired functioning (LFS) as outcome and Depression symptoms as second mediator) to .38 (in the model for males with impaired functioning (PDS) as outcome and PTSD symptoms as second mediator). Generally the total influence of the mediators was greatest concerning impaired functioning (PDS), followed by the level of physical complaints, aggressiveness, suicidal ideation, degree of stigmatization and impaired functioning (LFS). Taking mediation into account the remaining direct effects of abduction were in all models no longer relevant, except for the model incorporating impaired functioning (PDS) and Depression symptoms for males. Here partial mediation was present with abduction still being a significant predictor of functional impairment, though to a much lesser degree (β = .10, *p*<.05 versus β = .39).

## Discussion

In an epidemiological, household-based survey in IDP-camps and transition settlements in war-affected Northern Uganda we found that 43% of the children and young adults in the age-range between 12 and 25 reported a history of abduction by the rebel army, with 47% of them being held captive for one month or more. This finding shows that the often-cited (e.g. [5], [6], [11]) figure of 25.000 to 30.000 abducted Northern Ugandan children during the LRA insurgency is an underestimation. Other researchers have reported equally high abduction rates of 40% in a large-scale study with Northern Ugandans above age 18 [14], [15]. Although we could not replicate the excessive rates of psychopathology found in previous surveys mostly within selective samples, our data show that the level of psychopathology in former child soldiers is higher than in youths without abduction history and that they report impaired functioning. Further, our findings indicate that the former child soldiers' adjustment difficulties in the community are not caused by their history of abduction per se, but mediated by trauma exposure and the resulting psychopathology.

Consistent with a previous large-scale community survey in the area we found high rates of exposure to traumatizing events in the Northern Ugandan population [14]. Rates of trauma exposure were even higher in our sample, which can be attributed to the fact that our survey focused on children and young adults between 12 and 25, which resulted in the inclusion of higher numbers of children who had been abducted at a young age and held captive for longer time-periods.

Although a high-risk age-range was selected and reported traumatic exposure was exceptionally high in our survey, prevalence rates of PTSD were considerably lower than in most of the previous research in similar populations. We found a PTSD prevalence rate of 7% among non-abducted and 25% among formerly abducted individuals. Studies using convenience samples of former child soldiers found PTSD rates of 27% [16] and 33% [11] respectively and much above [10], [12], [13]. Population-based studies in Northern Uganda reported prevalence rates of probable PTSD of 51% and above for non-abducted and 67% and above for formerly abducted individuals [14], [15]. One major difference to these studies is that the present survey is the first investigation in the area applying locally validated instruments with cut-off scores that have been adapted to gold-standards. As the uncritical transfer of instruments from one population to the other risks errors in diagnoses, we assume that our findings are more precise and that previous figures of psychopathology might be inflated. Still, the extent of mental health impairment reached epidemic levels and alarmingly high rates of suicidal ideation were found, especially in the group of former child soldiers, with 34% presenting with suicide risk and 16% reporting high suicide risk.

### Prediction of Psychopathology

In line with numerous other studies (e.g. [14], [22]) our data strongly support the existence of a dose-effect of traumatic events on psychopathology. Beta weights were by far the highest for trauma exposure in the prediction of PTSD and Depression symptoms for the subgroup of former child soldiers as well as for the total war-affected sample. The fact that not all previous studies found this dosage-effect [10], [11] may be due to these surveys having studied highly selective groups of long-term abductees living in specialized rehabilitation facilities, who were more homogenous with regard to war-exposure.

In our sample, more than 57% of the former child soldiers reported that they had been forced to commit an atrocity at least once. Unlike Klasen et al. [11], we found that, independent of other traumatic experiences, these perpetration-events contributed to PTSD and Depression. Our results correspond to those reported by Hecker et al. [50], who found that perpetrating violence was positively related to symptoms of PTSD in forcibly recruited, but not in voluntary combatants. The finding that perpetration can be traumatizing has also been reported in war veterans [51] and research with Sierra Leonean child soldiers showed a strong association of killing and symptoms of depression and anxiety [17]. In addition to the personal threat connected to the forced exertion of atrocities, moral conflict and feelings of shame and guilt may arise and have a unique or aggravating impact on the mental health status of former child soldiers and their successful readjustment. Although the duration of abduction was highly correlated with trauma exposure, it had no independent influence on PTSD when exposure was controlled for. This is a common finding in former child soldier samples [10]–[12], [14].

In addition to trauma exposure, we found further predictors of psychopathology with smaller effect sizes. Female gender was an independent predictor of Depression, yet did not predict PTSD, which is consistent with Kohrt et al. [12] and Klasen et al. [11], but not Pham et al. [14]. Older age predicted Depression, which corresponds to the finding that Depression affects adults more than children. Consistent with previous studies [11], [12] we found a negative correlation between PTSD and economic status. However, the size of this relationship was comparatively small. Actively practicing religion seems to alleviate symptoms of Depression, but not PTSD in both, former child soldiers and the total sample of war-affected youth. Religiousity might help individuals to find meaning in what has happened, hope for a life after the present life of suffering and thus work against negative appraisal of the past, present and future. In former child soldiers the element of forgiveness of sins might play an additional role and mitigate feelings of guilt. The potential agent of regular praying is therefore more closely related to symptoms of Depression than to PTSD symptoms. The number of first-degree family members that died due to the war entered the analyses as an additional severe stressor that was not necessarily traumatic and played an independent role in predicting PTSD and Depression symptom load beyond traumatic exposure and socio-demographic variables.

### Mediation of impaired Readjustment

The results of the mediation analyses showed that twofold mediation was present. The multiple step multiple mediator models imply that the relationship between a history of abduction and maladjustment is predicted by the sequence of trauma exposure and psychopathology in both genders. This finding was robust across the composite variables as well as the single indicators of psychopathology and maladjustment, which included problems in daily functioning, suicide risk, illnesses, aggressiveness and stigmatization. The data showed that former child soldiers are especially vulnerable to developing psychopathology as well as experiencing difficulties in adjustment, not due to abductee-status per se, but due to the fact that chances of frequent exposure to traumatic events are higher during abduction and increase with abduction-duration [14], [52]. Similarly, Pham et al. [14] reported that their variable “having a problem returning home” was significantly associated with symptoms of PTSD and Depression. These findings are consistent with a perspective that PTSD and Depression are to a large extent responsible for multiple impairments even in war-torn communities that are affected by a multitude of hardships. Still, the fact that trauma exposure predicted maladjustment independent of psychopathology indicates that PTSD and Depression do not cover all physical and psychological damages caused by trauma exposure. Other psychological sequelae, as well as physical injuries caused by combat experience, malnutrition and infections might additionally contribute to disability.

Our analyses revealed that, contrary to the assumptions of aid agencies and the local population (e.g. [53]), abduction per se neither predicted aggressiveness nor stigmatization. The apparent positive relationships were fully mediated by traumatic exposure and psychopathology. Concerning aggressiveness, our results correspond with Maguen et al. and Betancourt et al. [17], [51], who reported a relationship between killing in the battlefield and showing violence or hostility back home. A positive relationship between psychopathology and attitudes favoring violence and hostility was stated by Vinck et al. [15] and Bayer et al. [10]. We speculate that aggressiveness might result from a cognitive bias to interpret events and conditions as threatening (e.g. [54]) as well as a learned tendency to react aggressively when threatened, a behavior that was rewarded in captivity. The latter hypothesis was supported by an additional multiple regression analysis that revealed that besides psychopathology, forced perpetration was the most important predictor of aggressiveness.

Furthermore, our results indicate that former child soldiers do not seem to experience stigmatization due to their status as ex-combatants as such. One important factor seems to be deviation from the norm in the form of psychological abnormality that causes the communities' negative attitude. A link between discrimination and symptoms of Depression and anxiety independent of war experiences has also been reported by Betancourt et al. [8]. Annan et al. [7] similarly state that poor acceptance by the community was associated with negative social behaviors and emotional distress. Phelan, Link, and Dovidio [55] propose three functions of stigma and prejudice: exploitation and domination (keeping people down), norm enforcement (keeping people in) and disease avoidance (keeping people away). The third aspect of Phelan et al.'s [55] typology seems to be mostly relevant in the Ugandan context and plays a role for both pathways to stigmatization. Humans fear to be “contaminated” by those who show abnormal behavior, a fact that might be especially relevant in cultures in which psychological symptoms are often connected to the world of spirits because people assume that evil spirits, or spirits of deceased people possess affected individuals and lead to overt symptoms like nightmares or flashbacks. These spirits and thus symptoms are suspected to be able to pass on from one individual to the other. Additionally perpetrators are believed to be possibly haunted by the spirits of the people they have killed.

### Limitations

Several limitations of this study indicate the need for further research. First of all, our data is cross-sectional which interferes with the interpretation of causality. Whereas the obvious temporal sequence of some variables can directly offer indications for the directions of some relationships, causality is unclear for other associations. Although the data fits well with the suggested mediation models, we cannot exclude the reversed causality for some elements of the model, in particular for aggressiveness and stigmatization. As suggested by Betancourt et al. [8], higher levels of stigmatization and aggressiveness might also influence psychopathology in the sense of perpetuating factors. Aggressiveness might be additionally influenced by stigmatization and vice versa. Secondly, like almost all studies with conflict populations, our study is based on self report. Potential recall and response biases might compromise the validity of the data and inflate the size of the correlations. Future studies should make use of peer-reports, objective community data as well as physiological indicators of psychopathology. Thirdly, although the respondents of the survey were chosen randomly from carefully selected clusters, the study cannot be representative of all former child soldiers. Our study represents a subset of those former Ugandan child soldiers and war-affected youths who voluntarily or involuntarily returned home and survived. Systematic differences to youths who died and those who still fight with the rebel army might exist and are not captured within this survey. Finally our models, though including many interrelating variables is presenting a simplified view of the complex interplay of war-related factors, psychological consequences and post-conflict readjustment. Pre- and post-conflict factors which were not assessed might influence traumatic exposure, as well as psychological reactions and later maladjustment and should be subject to further research.

## Conclusion

Taken together, our survey indicates that psychopathology is common among former child soldiers and that it is crucially associated with impaired functioning and readjustment. The results underline the need for a trauma-focused understanding of the consequences of severe stress related to war-exposure. These findings have important implications for the rehabilitation of former child soldiers. Although the group of formerly abducted children remains an especially vulnerable group due to the increased likelihood of frequent traumatic event exposure, the policy of offering psychosocial support to individuals based solely on the fact that they are former child soldiers may not effectively target those individuals in highest need. An efficient way to judge entry to therapeutic programs could be a brief event-load screening, which would be optimally combined with a short screening for core symptoms of PTSD and Depression. There is an undisputed need for programs designed to enable individuals to overcome disadvantages resulting from missed education in order to achieve self-sufficiency and reintegration (e.g. [56], [57]). However, our data indicate the additional need for treatment of PTSD and Depression in this population. Compared to previous estimations, a lower number of formerly abducted and war-affected individuals might need psychotherapeutic care to recover and readjust to post-conflict life and society. Recent studies showed that community-based psychotherapy programs are feasible and might be promising to treat mental health symptoms [58]–[60]. Further research should examine, whether treatment has a positive impact on child soldiers' readjustment, including stigmatization and hostile behavior or attitudes. Such studies could be informative to test the potential of psychotherapy to counteract the perpetuation of the cycle of violence in conflict-ridden areas.
